# Helix 12 stabilization contributes to basal transcriptional activity of PXR

**DOI:** 10.1016/j.jbc.2021.100978

**Published:** 2021-07-17

**Authors:** Ryota Shizu, Hikaru Nishiguchi, Sarii Tashiro, Takumi Sato, Ayaka Sugawara, Yuichiro Kanno, Takuomi Hosaka, Takamitsu Sasaki, Kouichi Yoshinari

**Affiliations:** Laboratory of Molecular Toxicology, School of Pharmaceutical Sciences, University of Shizuoka, Shizuoka, Japan

**Keywords:** coactivator, nuclear receptor, pregnane X receptor, transactivation domain, AF2, activation function 2, CAR, constitutive active/androstane receptor, DBD, DNA-binding domain, DMEM, Dulbecco's modified Eagle medium, DMSO, dimethyl sulfoxide, LBD, ligand-binding domain, PGC1α, peroxisome proliferator-activated receptor gamma coactivator-1α, PPAR, peroxisome proliferator-activated receptor, PXR, pregnane X receptor, RXR, retinoid X receptor, SRC, steroid receptor coactivator, VDR, vitamin D receptor, WT, wild-type

## Abstract

Pregnane X receptor (PXR) plays an important role in xenobiotic metabolism. While ligand binding induces PXR-dependent gene transcription, PXR shows constitutive transcriptional activity in the absence of ligands when expressed in cultured cells. This constitutive activity sometimes hampers investigation of PXR activation by compounds of interest. In this study, we investigated the molecular mechanism of PXR activation. In the reported crystal structures of unliganded PXR, helix 12 (H12), including a coactivator binding motif, was stabilized, while it is destabilized in the unliganded structures of other nuclear receptors, suggesting a role for H12 stabilization in the basal activity of PXR. Since Phe420, located in the loop between H11 and H12, is thought to interact with Leu411 and Ile414 to stabilize H12, we substituted alanine at Phe420 (PXR-F420A) and separately inserted three alanine residues directly after Phe420 (PXR-3A) and investigated their influence on PXR-mediated transcription. Reporter gene assays demonstrated that the mutants showed drastically reduced basal activity and enhanced responses to various ligands, which was further enhanced by coexpression of the coactivator peroxisome proliferator-activated receptor gamma coactivator 1α. Mutations of both Leu411 and Ile414 to alanine also suppressed basal activity. Mammalian two-hybrid assays showed that PXR-F420A and PXR-3A bound to corepressors and coactivators in the absence and presence of ligands, respectively. We conclude that the intramolecular interactions of Phe420 with Leu411 and Ile414 stabilize H12 to recruit coactivators even in the absence of ligands, contributing to the basal transcriptional activity of PXR. We propose that the generated mutants might be useful for PXR ligand screening.

Nuclear receptors are a group of transcription factors, most of which are activated by their cognate ligands that, and play pivotal roles in physiological and pathophysiological functions (1). Upon ligand binding, nuclear receptors form homodimers or heterodimers with other nuclear receptors, such as retinoid X receptor α (RXRα), in the nucleus and bind to their response elements on the promoter regions of target genes (2, 3). The receptors recruit transcriptional coactivators, such as steroid receptor coactivator 1 (SRC1, also known as NCOA1) or peroxisome proliferator-activated receptor gamma coactivator 1α (PGC1α), and induce the transcription of their target genes (4, 5).

Ligand binding to the ligand-binding domain (LBD) of nuclear receptors constitutes the initial step in target gene regulation. All nuclear receptor LBDs share the same conserved 12 α-helix architecture. In this context, the C-terminal helix 12 (H12), termed activation function 2 (AF2), in the LBDs plays a key role in gene regulation by recruiting coregulators. Structural studies have shown that the configuration of AF2 alters depending on ligand binding, and this agonist binding-dependent conformational alteration enables the receptor to recruit its coactivators (6, 7). In contrast, antagonist binding to the LBD prevents AF2 from adopting the active stabilized conformation and induces the recruitment of corepressors.

Pregnane X receptor (PXR), encoded by *NR1I2* in humans, is a nuclear receptor that is highly expressed in the liver and activated by numerous compounds including drugs, food ingredients, and pesticides. Ligand binding to PXR causes it to translocate from the cytoplasm to the nucleus to induce the transcription of genes encoding drug-metabolizing enzymes such as cytochrome P450s and drug transporters (8, 9). Since PXR activation enhances xenobiotic metabolism and disposition, it may cause drug–drug or drug–food interactions. Therefore, PXR activation by exogenous chemicals has been extensively studied for drug development and food and chemical safety (10, 11).

Traditionally, chemical activation of PXR is assessed by cell-based reporter gene assays and/or by determining the mRNA levels of PXR target genes, such as *CYP3A4*, in hepatocytes. More recently, *in vitro* high-throughput screening methods using recombinant proteins, including time-resolved fluorescence resonance energy transfer (TR-FRET) (12, 13), fluorescence polarization/anisotropy (14), isothermal titration calorimetry (15), hydrogen-deuterium exchange (16, 17), differential scanning fluorometry (18), and surface plasmon resonance (19), have been applied. Most of these recently developed screening systems are based on the ligand-binding-dependent conformational changes of the LBD, especially the conformational changes of AF2. For high-throughput screening, understanding the conformational changes in ligand-activated nuclear receptors in detail is required.

Although PXR is a ligand-activated nuclear receptor, it is reported that PXR has constitutive transcriptional activity regardless of ligand binding, and its ligands regulate the localization of PXR from the cytoplasm to the nucleus (8, 20). It is well known that transient expression of PXR in cultured cells induces constitutive nuclear localization and upregulates the transcription of target genes in the absence of any ligand (21). This ligand-independent basal activity is not observed in other ligand-activated nuclear receptors, such as retinoic-acid-activated RXRα, peroxisome proliferator-activated receptor gamma (PPARγ), and vitamin D receptor (VDR) (22, 23, 24). Since basal PXR activity sometimes interferes with its sensitivity to ligand-dependent activation in reporter assays, reduction of the basal activity is needed for high-throughput screening of PXR ligands. In addition, a mechanistic understanding of this basal activity may provide insight into how ligands control nuclear receptor-mediated gene expression.

RXRα activation is ligand-dependent and the crystal structure of unliganded RXRα has been reported (25, 26). Compared with representative ligand-activated nuclear receptors, the coactivator-binding AF2 domain of unliganded RXRα is located far from the stable position to recruit coactivators.

Here, using human PXR, we demonstrate the mechanism of the constitutive transcriptional activity of PXR and report the construction of PXR mutants that drastically suppress basal activity while maintaining high ligand sensitivity.

## Results

### Interaction of Phe420 with Leu411 and Ile414 contributes to the constitutive activity of PXR

**Figure 1:**
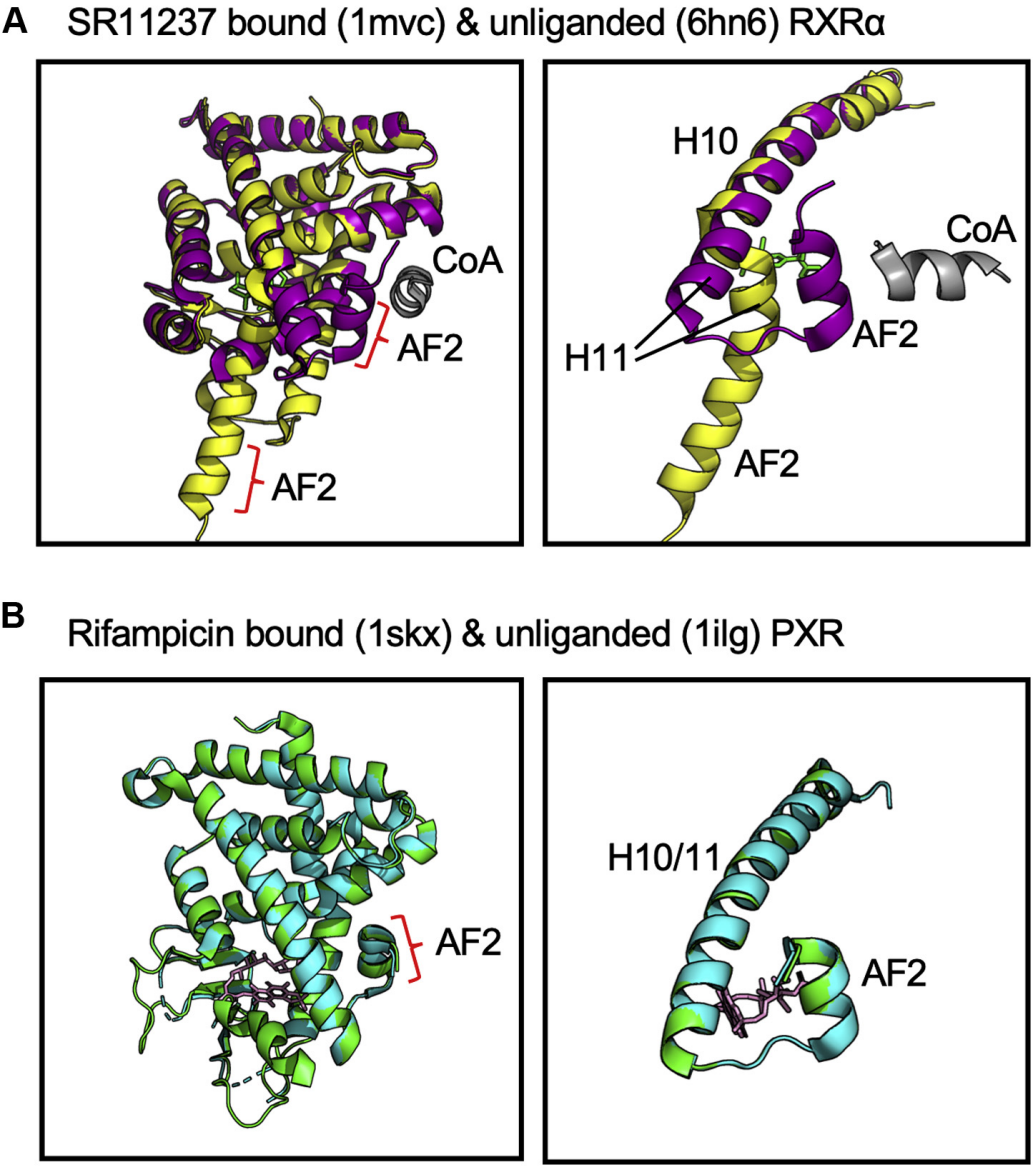
**3D structures of ligand-bound and unbound nuclear receptor LBDs.***A*, superimposed crystal structures of RXRα LBD with its ligand SR11237 (1mvc, *purple*) and unliganded RXRα LBD (6hn6, *yellow*). *Left*, a view including the entire LBD structure. *Right*, a close-up view of H10 to H12; SR11237 (*green*) binding repositions H11 to be continuous after H10 and H12 packs against the core of the LBD. CoA, coactivator peptide (*gray*). *B*, superimposed crystal structures of PXR LBD with its ligand rifampicin (1skx, *cyan*) and unliganded PXR LBD (1ilg, *green*). *Left*, a view including the entire LBD structure. *Right*, a close-up view of H10 to H12; rifampicin (*pink*) binding does not alter the conformation of H11 to H12.

As shown in Figure 1, the crystal structure of the unliganded RXRα LBD (PDBID; 6HN6) (26) suggest that there are several conformational differences from the SR11237-bound RXRα LBD (PDBID; 1MVC) (25). Upon ligand binding, major structural changes were observed in H3 and H11 to H12. The position of H11 to H12 in the unliganded state was obviously different from that in the ligand-bound state (Fig. 1*A*). In the apo structure, H11 to H12 is located in a position where coactivators are unable to bind to the receptor (6, 7), and H11 and H12 obstruct the N-terminal portion of H3, bending toward the interior of the ligand-binding pocket (Fig. S1*A*) (6, 26). Upon ligand binding, H11 pulls out of this pocket and is positioned continuously with H10 with the N-terminal portion of H3 now bending toward the ligand (6, 26). Subsequently, AF2 reorients to a position where coactivators can contact the LBDs. Nolte *et al.* (27) reported that AF2 in the crystal structures of the unliganded PPARγ LBD is also located slightly distant from of its position in ligand-bound PPARγ. In contrast, the conformation of the AF2 domain in the reported crystal structures of the unliganded PXR LBD (PDBIDs; 7AX8, 4J5W, 3CTB, and 1ILG) (14, 28, 29, 30) was found to be different from other nuclear receptors. In these cases, the AF2 domain was located in the same position as the reported crystal structures of ligand (rifampicin or SR12813)-bound LBDs with a coactivator peptide (PDBIDs; 1SKX, 3HVL, 1NRL, and 4J5X) (14, 29, 31, 32). These observations had raised a possibility that the constitutive basal activity of PXR can be attributed to AF2 being in the stabilized position independent of ligand presence.

**Figure 2:**
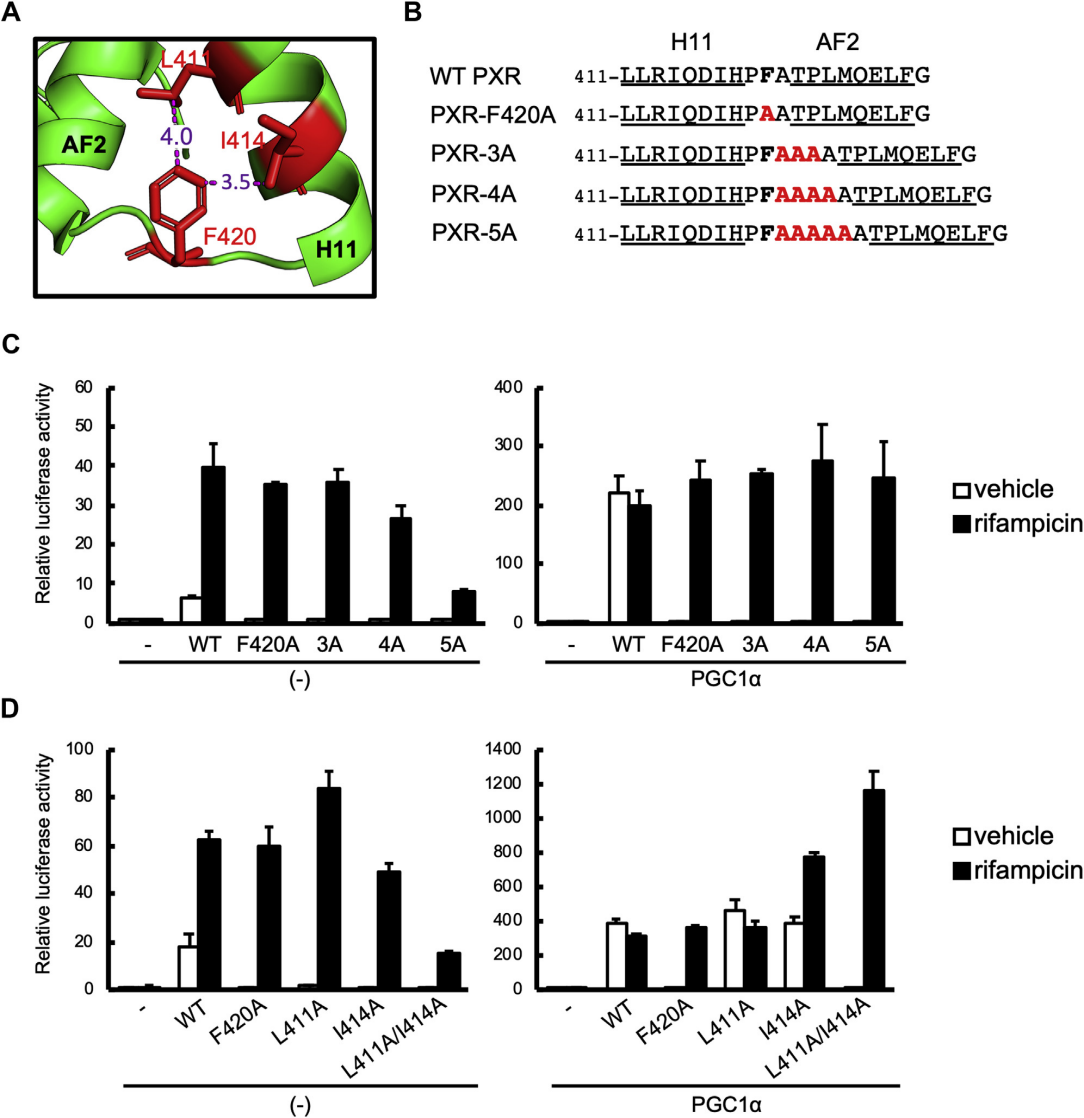
**The influence of the modified PXR H11 to H12 region on its transactivation.***A*, side chains from H11 to H12, including Leu411, Ile414, and Phe420, are mapped in the unliganded PXR structure (1ilg). *B*, the amino acid sequences of WT and mutant PXR. H11 and H12 sequences are *underlined*. *C* and *D*, reporter gene assays were performed in COS-1 cells with the reporter construct containing the promoter for *CYP3A4* (p3A4-pGL3) and expression plasmid for WT PXR (WT), PXR-F420A (F420A), PXR-3A (3A), PXR-4A (4A), PXR-5A (5A), PXR-L411A (L411A), PXR-I414A (I414A), or PXR-L411A/I414A (L411A/I414A) in combination with or without an expression plasmid for PGC1α. Cells were treated with rifampicin (10 μM) or vehicle (0.1% DMSO) for 24 h, then reporter activity was determined. Data are shown as the mean of the relative reporter activities of four wells in each group to vehicle-treated cells without PXR and PGC1α. Error bars represent the standard deviations.

From the available PXR LBD structures (14, 28, 29, 30), Phe420, located in the loop between H11 and H12, appears to interact with Leu411 and Ile414 in H11 *via* van der Waals interactions at distances of 4.0 Å and 3.5 Å (PDBID; 1ILG) (30), respectively (Fig. 2*A*). Since these interactions might prevent conformational flexibility of the AF2 domain, we substituted alanine for phenylalanine at this position (PXR-F420A). In addition, 3 to 5 alanine residues were inserted after Phe420 (PXR-3A, PXR-4A, and PXR-5A, respectively) to expand the loop between H11 and AF2 (Fig. 2*B*).

Either wild-type (WT) or mutant PXR was transiently expressed in COS-1 cells, and reporter gene assays were conducted to determine their transcriptional activity (Fig. 2*C*). As expected, the expression of WT PXR increased reporter activity even in the absence of the ligand rifampicin, while rifampicin treatment further increased reporter activity. In contrast, in all mutants described above, the basal activity was completely abolished, while rifampicin-induced activity was comparable to WT, except for PXR-5A, which showed diminished ligand inducibility. These results suggest that the Phe420-containing loop is associated with PXR constitutive activity.

We then investigated the influence of coactivator coexpression on PXR transcriptional activity. Since we previously demonstrated that PGC1α was the most effective coactivator for PXR-dependent gene transcription among the five reported PXR coactivators (33), we coexpressed PGC1α in this system (Fig. 2*C*). In the absence of a ligand, PGC1α coexpression increased reporter activity in WT PXR, but not in the mutants. Rifampicin treatment enhanced the activity of all mutants to levels comparable to unliganded WT PXR, but did not further increase the activity of WT PXR. Overexpression of another coactivator, SRC-1, also increased the reporter activity of unliganded WT PXR, but not PXR-3A (Fig. S2). Rifampicin treatment increased the activity of both WT and PXR-3A (Fig. S2). These results suggest that coactivators can bind to the mutants and fix the AF2 domain at the transcriptionally active position in a ligand-dependent manner.

Leu411 and Ile414 in H11 were predicted to interact with Phe420 *via* van der Waals interactions. Hence, these residues were individually (PXR-L411A and PXR-I414A) or simultaneously (PXR-L411A/I414A) mutated, and the transcriptional activity of the mutants was investigated (Fig. 2*D*). Both PXR-L411A and PXR-I414A showed reduced basal activity as PXR-F420A, whereas the mutants were responsive to rifampicin treatment, with a maximum activity comparable to WT PXR. When PGC1α was coexpressed, both PXR-L411A and PXR-I414A exhibited equivalent constitutive activity to WT PXR under unliganded conditions, but the PXR-L411A/I414A double mutant showed negligible basal activity, suggesting that Phe420 interactions with both L411A and I414A are important for fixing the AF2 domain in a coactivator-binding position. Gln415 in H11 was also suggested to participate in an interaction motif with Phe420 (Fig. S3*A*). As expected, the Q415A mutation also prevented basal activity (Fig. S3*B*).

The reported crystal structures of PXR suggested that rifampicin contacts His407, Arg410, Leu411, Ile414, and Phe420 in H11 to H12, and SR12813 contacts His407, Leu411, Phe420, Met425, and Phe429 (Fig. S4*A*). It is known that PXR ligands stabilize H12 through interactions with Met425 and Phe429 in AF2 (15, 17, 34). These residues bind ligands to allow H12 to stabilize as a cover over the ligand-binding pocket for interactions with coactivators. Simultaneous mutation of these two residues clearly reduced both basal and ligand-induced transcriptional activity of both WT PXR and PXR-F420A, even in the presence of coexpressed PGC1α (Fig. S4*B*). This result suggests that these mutations prevented H12 from being packed in a stable position to interact with coactivators.

Next, we investigated the subcellular localization of green fluorescence protein (GFP)-tagged WT PXR, PXR-3A, PXR-F420A, PXR-L411A, PXR-I414A, and PXR-L411A/I414A in COS-1 cells. The results showed that all the mutants, as well as WT PXR, accumulated in the nucleus regardless of rifampicin treatment, suggesting that these mutations did not affect subcellular distribution (Fig. S5).

### Influence of Phe420-related mutations on coregulator recruitment of PXR

**Figure 3:**
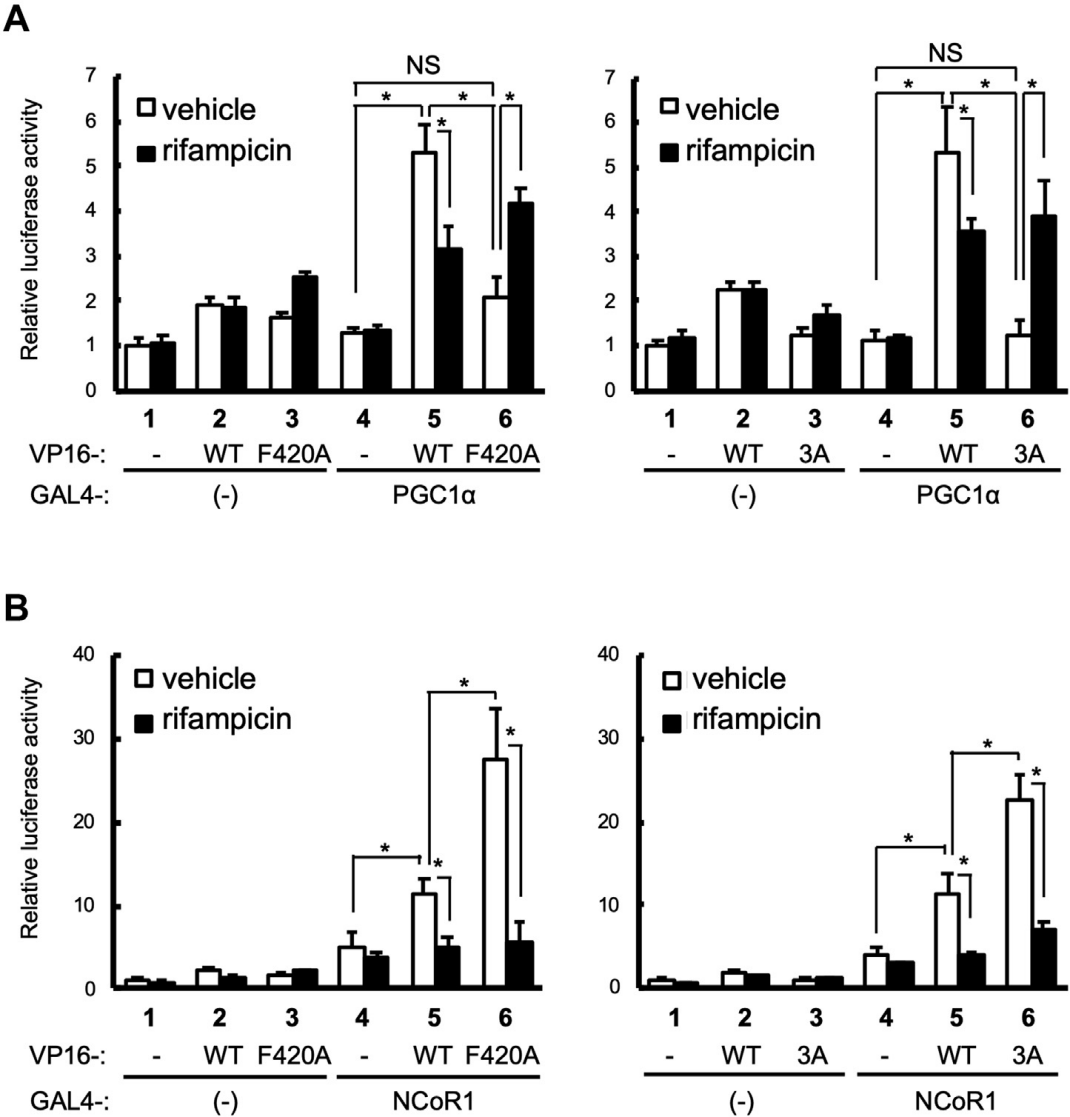
**Interaction between PXR and cofactors in mammalian two-hybrid assays.***A* and *B*, mammalian two-hybrid assays were performed in COS-1 cells with pGL4.31, pFN11A expressing GAL4 or GAL4 fused with PGC1α or NCoR1, and pFN10A expressing VP16 or VP16 fused with WT PXR (WT), PXR-F420A (F420A), or PXR-3A (3A). Cells were treated with vehicle (0.1% DMSO) or rifampicin (10 μM) for 24 h, and then reporter activity was determined. Data are shown as the mean of the relative reporter activities of four wells in each group to vehicle-treated cells without PXR and PGC1α. Error bars represent the standard deviations. Statistical analyses were performed for the indicated combinations with Bonferroni’s correction (∗*p* < 0.05; NS, not significant).

To investigate the influence of the Phe420-related mutations on the ligand-dependent recruitment of coactivators and corepressors on AF2, mammalian two-hybrid assays were conducted with the nuclear receptor interacting motif (LXXLL) of PGC1α fused to the GAL4 DNA-binding domain (DBD) and PXR fused to the VP16 transactivation domain (Fig. 3*A*). Binding of the PGC1α LXXLL motif to WT PXR was observed in the absence of rifampicin (columns 4 *versus* 5, open bars). Although the reason is unknown, rifampicin treatment diminished this interaction. As expected, unliganded PXR-F420A and PXR-3A showed insignificant or no interaction with PGC1α (columns 4 *versus* 6, open bars), respectively, while significant binding was observed with rifampicin treatment (columns 4 *versus* 6, closed bars). The same results were obtained for SRC1 (Fig. S6).

Since AF2 at the destabilized position binds to corepressors (35), corepressor binding was also investigated by mammalian two-hybrid assays (Fig. 3*B*). While unliganded WT PXR interacted with NCoR1, rifampicin treatment prevented this interaction (column 5). Both PXR-3A and PXR-F420A showed increased interactions with NCoR1 compared with WT PXR, and rifampicin treatment blocked this interaction (column 6). These results suggest that WT PXR could bind to both coactivators and corepressors with different binding affinities in an unliganded state and that ligand binding decreases corepressor binding.

Taken together, these *in vitro* binding assay results suggest that Phe420-related mutations increase the flexibility of AF2 to weaken binding to coactivators, while these mutations enhance binding to corepressors in the absence of ligands.

### Influence of Phe420-related mutations on ligand-dependent PXR transactivation

**Figure 4:**
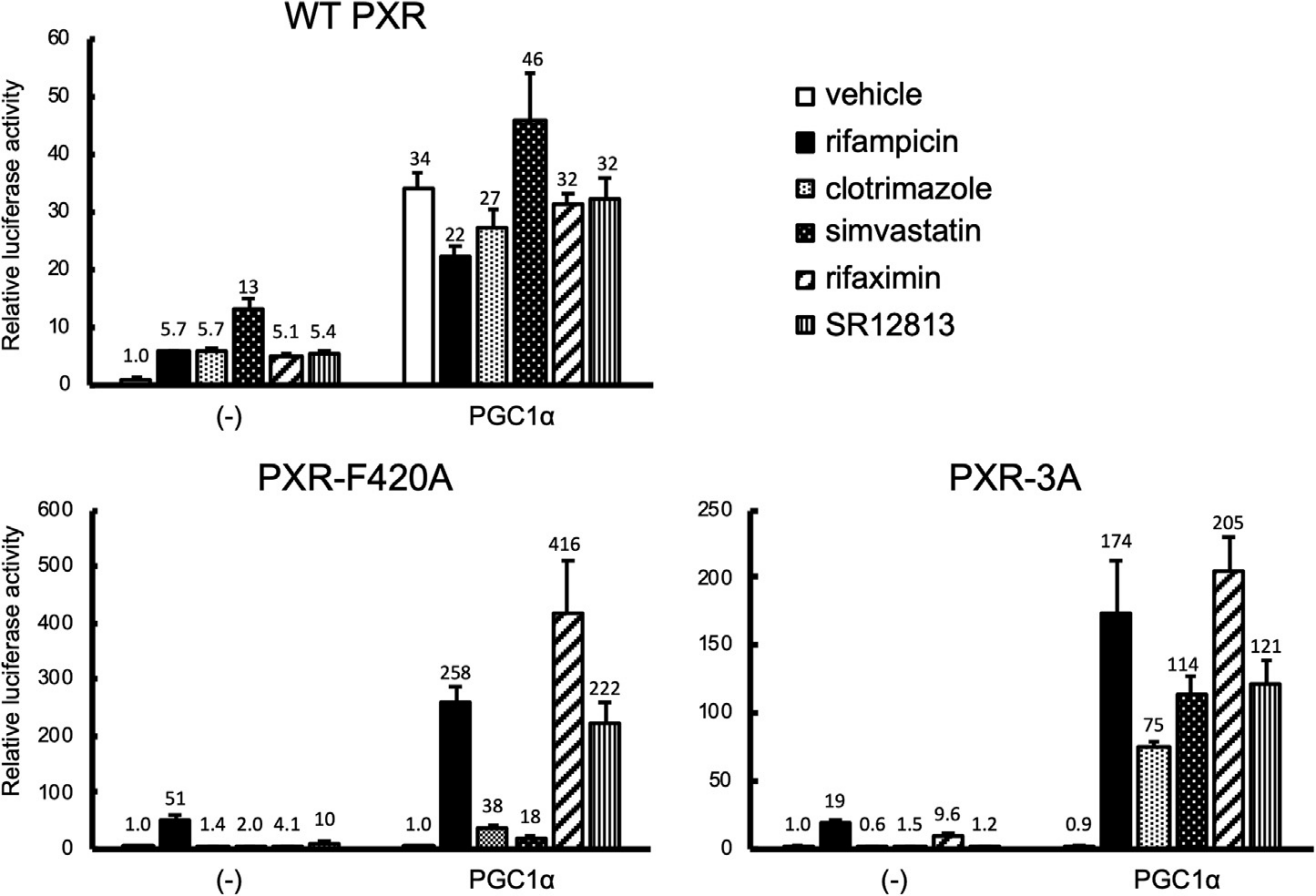
**Activation of WT and mutant PXR by typical PXR ligands.** Reporter gene assays were performed in COS-1 cells with the reporter construct containing the promoter for *CYP3A4* (p3A4-pGL3) and expression plasmids for WT PXR (WT), PXR-F420A (F420A), or PXR-3A (3A) in combination with or without the expression plasmid for PGC1α. Cells were treated with vehicle (0.1% DMSO), rifampicin (10 μM), clotrimazole (10 μM), simvastatin (10 μM), rifaximin (10 μM), or SR12813 (10 μM) for 24 h, then reporter activity was determined. Data are shown as the mean of the relative reporter activities of four wells in each group to vehicle-treated cells without PGC1α. Error bars represent the standard deviation. The numbers above the column indicate the relative reporter activity to vehicle-treated cells without PGC1α expression.

To assess the influence of Phe420-related mutations on transcriptional activation induced by known PXR ligands other than rifampicin, reporter assays were conducted with WT PXR, PXR-3A, and PXR-F420A and several ligands at 10 μM (Fig. 4). In this system, the reporter activity of WT PXR was increased 5- to 13-fold by ligand treatment in the absence of PGC1α. As demonstrated above, PGC1α coexpression induced reporter activity of unliganded PXR while no additional ligand-dependent induction was observed. In the absence of PGC1α, rifampicin showed the strongest activation of both PXR-F420A and PXR-3A among the ligands tested. SR12813 and rifaximin increased activity by approximately tenfold for both PXR-F420A and PXR-3A, while clotrimazole and simvastatin showed no or minimal activation, respectively, of the PXR mutants in the absence of PGC1α. In contrast, PGC1α coexpression clearly increased the sensitivity of these mutants to these ligands to varying degrees depending on the mutant and ligand (*e.g.*, 18-fold with simvastatin to 416-fold with rifaximin for PXR-F420A and 75-fold with clotrimazole to 205-fold with rifaximin for PXR-3A). These results suggest that these mutations increase sensitivity to various PXR ligands in the presence of PGC1α.

**Figure 5:**
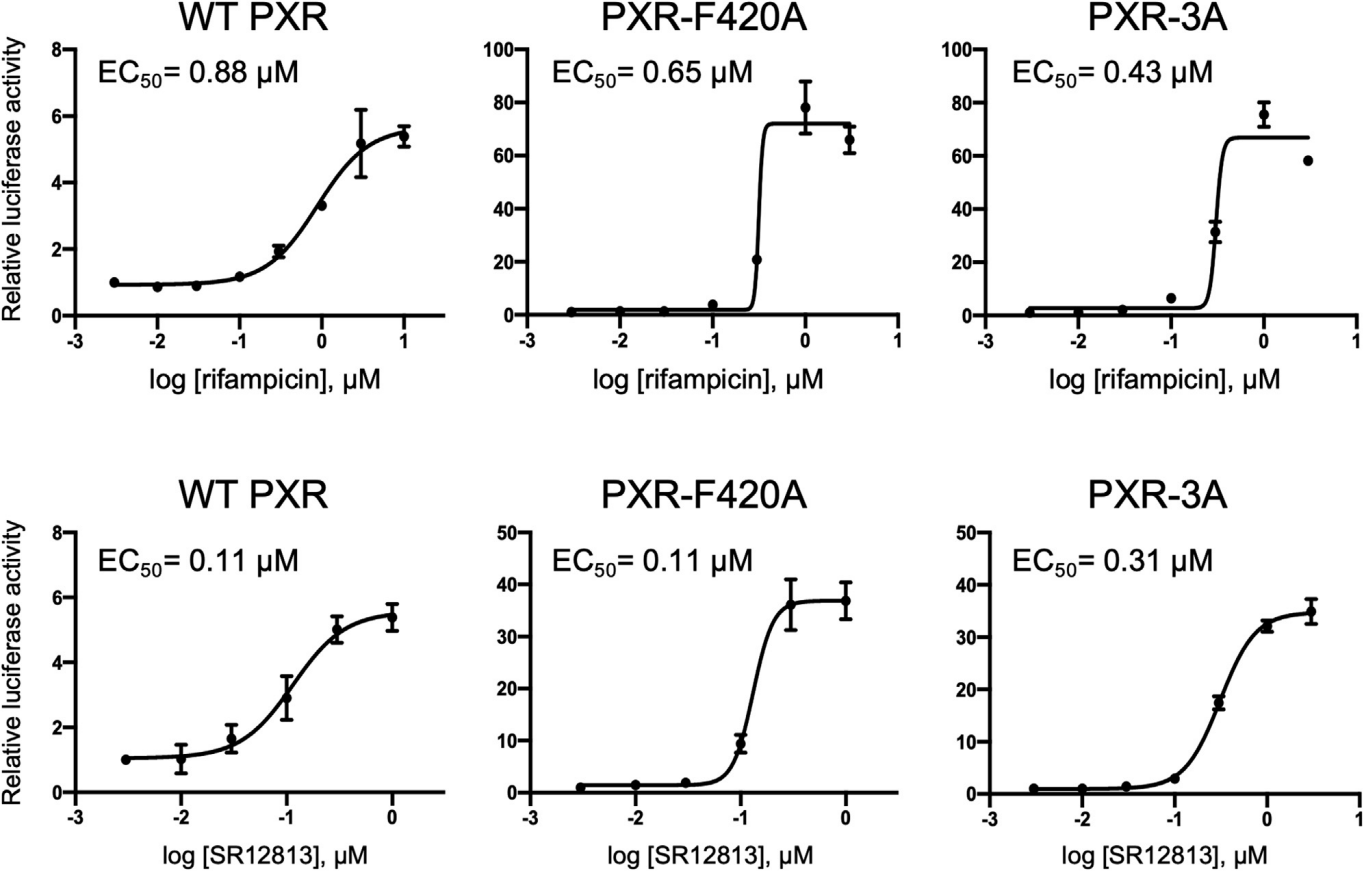
**Dose-dependent activation of WT and mutant PXR by ligands.** Reporter gene assays were performed in COS-1 cells with the reporter construct containing the promoter for *CYP3A4* (p3A4-pGL3) and expression plasmids for WT PXR, PXR-F420A, or PXR-3A in combination with the expression plasmid for PGC1α. Cells were treated with vehicle (0.1% DMSO), rifampicin, or SR12813 at the indicated doses for 24 h. Then, the reporter activity was determined and EC_50_ values were calculated using GraphPad Prism. Data are shown as the mean of the relative reporter activity of the four wells in each group to vehicle-treated cells. Error bars represent standard deviation.

To further characterize the increase in sensitivity, dose-dependent activation of the mutants with rifampicin and SR12813 was investigated in the presence of PGC1α, and EC_50_ values were calculated (Fig. 5). Although the maximum activities (*i.e.*, E_max_ values) were different, the EC_50_ values of rifampicin- and SR12813-dependent activation of PXR-F420A and PXR-3A were comparable to WT PXR.

Knowing the EC_50_ values, we also tested the ligands at lower concentrations (0.1 and 1 μM) in the presence or absence of PGC1α (Fig. S7). Without PGC1α, 0.1 μM SR12813 treatment induced reporter activity (2.4-fold) of WT PXR but not PXR-F420A. In addition, weak induction was observed with clotrimazole, simvastatin, and rifaximin at 1 μM for WT PXR but not for PXR-F420A in the absence of PGC1α. When PGC1α was coexpressed, PXR-F420A responded to the ligands at the lower concentrations to various extents.

Taken together, these results suggest that the F420 mutation might increase the degree of ligand-induced transactivation despite that the PXR-F420A mutant possibly has lowered ligand-binding affinity without PGC1α on the ligand.

### Influence of antagonists on ligand-dependent activation of PXR mutants

**Figure 6:**
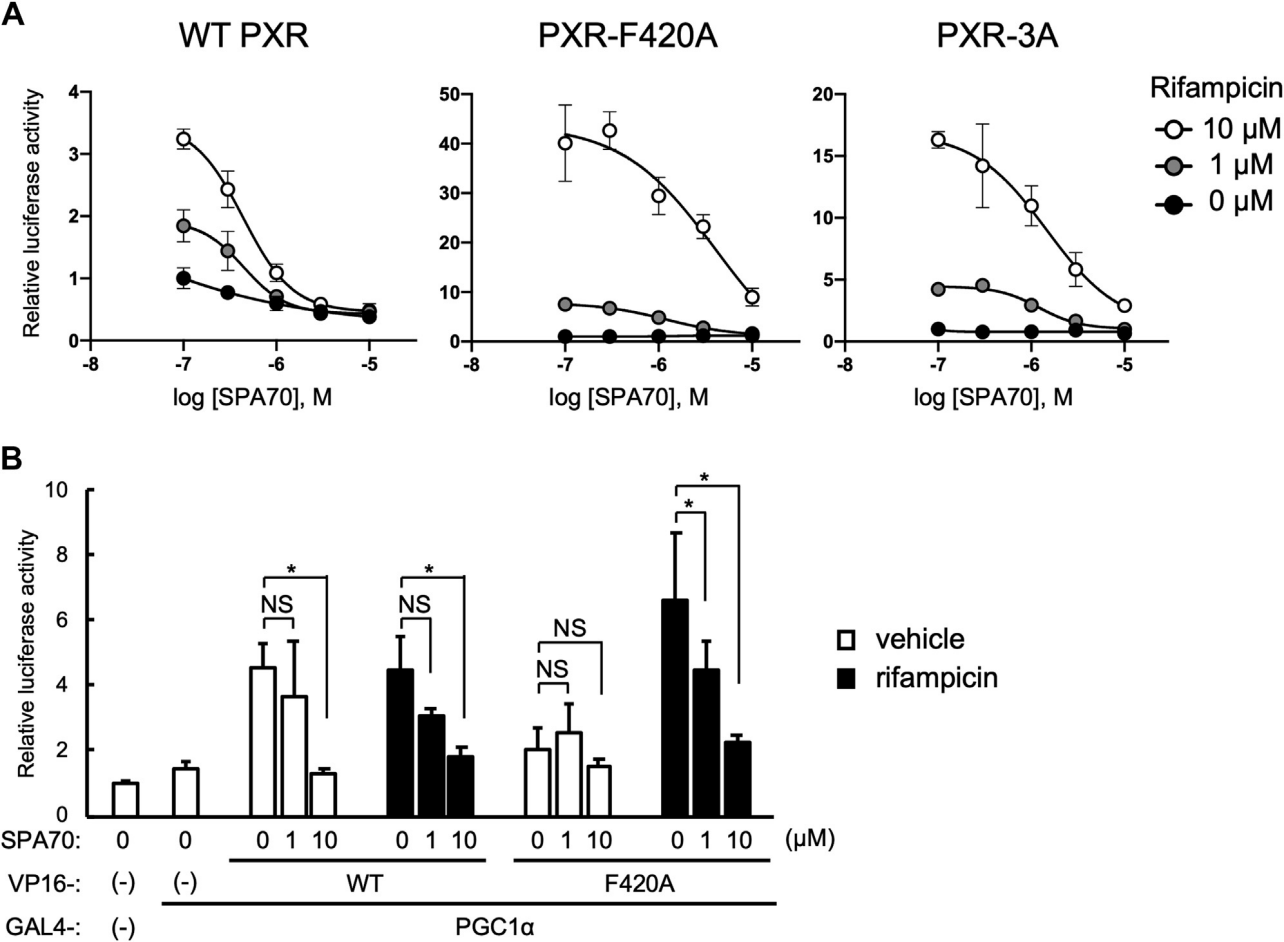
**Influence of PXR antagonists on WT and mutant PXR.***A*, reporter gene assays were performed in COS-1 cells with the reporter construct containing the promoter for *CYP3A4* (p3A4-pGL3) and the expression plasmid for WT PXR, PXR-F420A, or PXR-3A in combination with the expression plasmid for PGC1α. Cells were treated with rifampicin and/or SPA70 at the indicated doses for 24 h. Then, the reporter activity was determined and IC_50_ values were calculated using GraphPad Prism. Data are shown as the mean of the relative reporter activity of four wells in each group to vehicle-treated cells. Error bars represent the standard deviations. *B*, mammalian two-hybrid assays were performed in COS-1 cells with pGL4.31, pFN11A expressing GAL4 (−) or GAL4 fused with PGC1α (+), and pFN10A expressing VP16 (−) or VP16 fused with WT PXR (WT) or PXR-F420A (F420A). Cells were treated with vehicle (0.1% DMSO) or rifampicin (10 μM) and/or SPA70 (1 or 10 μM) for 24 h, and then reporter activity was determined. Data are shown as the mean of the relative reporter activity of four wells in each group to vehicle-treated cells expressing GAL4 and VP16 only. Error bars represent the standard deviations. Statistical analyses were performed for the indicated combinations with Bonferroni’s correction (∗*p* < 0.05; NS, not significant).

Finally, the influence of these mutations on response to the PXR antagonist SPA70 was investigated (Fig. 6*A*). SPA70 is reported to reduce AF2 stability by disrupting its interactions with either Phe429 or Leu428 in AF2 and/or preventing AF2 from being positioned for coactivator recruitment (17, 35). SPA70 treatment almost completely blocked rifampicin-induced transactivation of WT PXR, PXR-F420A, and PXR-3A. The IC_50_ values for activation by 10 μM rifampicin were 0.47 μM, 4.08 μM, and 1.46 μM, for WT PXR, PXR-F420A, and PXR-3A, respectively. Similar results were obtained with the antagonist ketoconazole (Fig. S8).

To confirm the effects of the antagonists, mammalian two-hybrid assays were performed (Fig. 6*B*). As expected, SPA70 treatment prevented the ligand-dependent interaction of PXR-F420A with PGC1α, as well as the interaction of both liganded and unliganded WT PXR with PGC1α. These results indicate that the mutants are responsive to antagonists and can distinguish between agonists and antagonists.

## Discussion

The reported crystal structures of ligand-bound nuclear receptor LBDs, such as for RXRα, suggest that the AF2 domains are stabilized at the position where they interact with coactivator peptides (6, 7). However, for PXR, the crystal structures for the unliganded LBD suggest that the AF2 domain for this nuclear receptor is oriented at this position even in the absence of ligands, which confers a constitutive level of PXR transcriptional activity (14, 28, 29, 30). Our present results indicate that the interaction between Phe420 and Leu411/Ile414 might be the reason for AF2 stabilization and basal activity of PXR. The substitution of Phe420 with alanine, as well as the mutation of both Leu411 and Ile414 to alanine, suppressed basal activity. These results imply that the flexibility of the PXR AF2 is less than other nuclear receptors because of these interactions between Phe420 and Leu411/Ile414.

Mammalian two-hybrid assays showed that Phe420-related mutants bound to coactivators in a ligand-dependent manner. However, increased binding to the corepressor NCoR1 was observed for these mutants in the absence of ligands, and ligand treatment decreased this interaction. Corepressor binding was observed when AF2 was not oriented in the stabilized position where the coactivator interacted with AF2 (35). In addition, antagonist binding to a ligand-binding pocket causes AF2 destabilization to favor its interaction with corepressors (36, 37). Considering these observations, the present results strongly suggest that these mutations alter the position of AF2 from the stabilized to a destabilized position, increasing the flexibility of the AF2 domain to reduce coactivator binding, and increase corepressor binding.

Phe420 was reported as the main residue that interacts with ligands in the PXR LBD (38, 39, 40) (Fig. S4); the PXR-F420A mutant was not activated by ligands (41). Thus, it was anticipated that PXR-F420A could not bind ligands. In reporter assays with 10 μM ligands and without PGC1α coexpression, no or very weak activation by ligands other than rifampicin was observed for PXR-F420A. The ligand concentrations used in this study are well above the EC_50_ values for the activation of WT PXR by rifampicin and SR12813. However, in the presence of PGC1α, PXR-F420A responded to all ligands used in this study, and the EC_50_ values for the activation of the mutant by rifampicin and SR12813 were comparable to WT PXR. These results indicate that, although PXR-F420A may have lowered ligand-binding affinity, the presence of PGC1α might offset the low affinity of the mutant for chemical activation.

Although PXR-F420A responded to all ligands, only partial stimulation by PGC1α coexpression was observed with some ligands, including clotrimazole and simvastatin. However, when 150 chemical compounds were tested for the activation of WT PXR and PXR-F420A in the presence of PGC1α, we found significant overlap of the compounds that activated both WT PXR and PXR-F420A (unpublished data). These results suggest that a reporter assay system using the PXR-F420A mutant (and/or PXR-3A) in combination with PGC1α is useful for evaluating the chemical activation of PXR.

Based on PXR crystal structures (31, 32), Leu411 and Ile414 are residues that are in close contact with ligands such as rifampicin or SR12813. Mutation of these residues may affect ligand-binding affinity. However, rifampicin treatment clearly induced reporter activity in L411A and I414A mutants. Since mutation of Leu411 and/or Ile414 prevented basal activity, the interaction between Phe420 and Leu411 and Ile414 is clearly important for the stabilization of this C-terminal helical motif.

In addition, Gln415 in H11 and Met425 in AF2 are also expected to interact with Phe420 (Fig. S3*A*). Gln415 may interact with the amide NH of Phe420 *via* hydrogen bonding with the side chain C=O. Met425 may form van der Waals interactions within a distance of 3.5 Å. The stabilization of these C-terminal helices may be caused by these intramolecular interactions between these residues.

It is well known that species differences in PXR ligands result from differences in residues in PXR LBDs (42). Constitutive transcriptional activity is commonly observed for PXR in various species, including mice, rats, and humans. Since Phe420, Ile414, and Leu411 are conserved among these species, the interactions of these residues might be a common underlying mechanism of PXR basal activity.

Coexpression of PGC1α with PXR-F420A or PXR-3A clearly increased fold-induction values of reporter activity in response to ligand treatment. However, as shown in Figure 4, the induction profiles by various ligands of these mutants with PGC1α were clearly different from WT PXR; simvastatin activated WT PXR more than rifampicin, while the simvastatin-dependent activation was less than rifampicin-dependent activation for the PXR mutants. These results imply that the contribution of each coactivator to ligand-dependent activation differs depending on the ligand. Namely, PGC1α may play a significant role in rifampicin-dependent transcription, but less so in simvastatin-dependent transcription. Thus, the PXR mutants might help study the association between PXR ligands and coactivators.

Ligand screening of PXR by high-throughput reporter assay-based methods is sometimes conducted to evaluate drug–drug interactions or chemical safety. For example, in the Tox21 project conducted by public research institutes in the United States, 10,000 chemicals were tested at 15 concentrations against a panel of nuclear receptors, including PXR, by reporter or one-hybrid assays (43). The reporter assay with PXR-F420A showed clear ligand-dependent activation and could be a suitable system for high-throughput screening of PXR ligands.

Recently developed *in vitro* evaluation systems, such as TR-FRET, detect the interaction between nuclear receptors and coactivators of interest based on ligand-dependent conformational changes. Since PXR-F420A and/or PXR-3A clearly prevented basal activity and were obviously upregulated by ligand binding, the mutants might be suitable for such *in vitro* systems. The applicability of these mutants to these *in vitro* high-throughput screening needs to be evaluated in future studies.

Similar to PXR, the nuclear receptor constitutive active/androstane receptor (CAR) also exhibits basal activity in the absence of ligands. Since the crystal structure of unliganded CAR has not been reported, the orientation of AF2 in unliganded CAR is unclear. However, because PXR and CAR have shorter loops between H11 and H12 than other nuclear receptors, the constitutive activity of CAR might also be caused by low flexibility and tight packing conditions of the AF2 domain. In fact, the insertion of three alanine residues between H11 and H12 was shown to reduce basal activity by preventing coactivator interaction (44, 45). These results suggest that the position and flexibility of AF2 under unliganded conditions might determine the basal transcriptional activity of not only PXR and CAR but also other nuclear receptors.

Recent studies have shown that the role of PXR extends far beyond the regulation of drug metabolism. Its activation regulates hepatic energy metabolism (46), inflammation, and apoptosis (47, 48). PXR also plays a role in the regulation of cancer development (49, 50). The ligand-sensitive activated mutants might be useful for characterizing new PXR activators to study the biological functions of PXR.

## Experimental procedures

### Reagents

Clotrimazole, ketoconazole, rifampicin, rifaximin, SR12813, and simvastatin were purchased from Sigma-Aldrich. SPA70 was obtained from Axon Medchem. Oligonucleotides were commercially synthesized by Macrogen. Restriction enzymes were purchased from New England Biolabs. All other reagents were obtained from Fujifilm Wako Pure Chemical or Sigma-Aldrich, unless otherwise indicated.

### Plasmid preparation

The human PXR (hPXR) pTarget plasmid and p3A4-pGL3 have been reported previously (33). hPXR-pFN10A was constructed by inserting the amplified hPXR cDNA into pFN10A (Promega) at the *Sgf*I/*Pme*I sites. The pFN11A-based expression plasmid for the PGC1α-LXXLL motif (EAEEPSLLKKLLLAPANTQ) fused to the GAL4 DBD protein (PGC1α-LXXLL-pFN11A) was constructed previously (51). phRL-TK, phRL-CMV, and pFN21A were purchased from Promega. All mutations or insertions were generated using PrimeSTAR Max DNA Polymerase (Takara Bio) and confirmed by sequencing.

### Cell cultures

COS-1 cells were cultured in Dulbecco's Modified Eagle medium (DMEM, Fujifilm Wako Pure Chemical) supplemented with 10% fetal bovine serum (FBS, GE Healthcare), MEM nonessential amino acids, and antibiotic-antimycotic (Thermo Fisher Scientific). Twenty-four hours after seeding, the culture medium was replaced with prewarmed DMEM without FBS, and plasmids were transfected with Lipofectamine 3000 (Thermo Fisher Scientific) according to the manufacturer’s instructions.

### Reporter gene assays

COS-1 cells were transfected with the p3A4-pGL3 expression plasmid and the *Renilla* luciferase-expressing plasmid phRL-TK using Lipofectamine 3000 and treated with vehicle (0.1% or 0.2% dimethyl sulfoxide; DMSO) or drugs in serum-free DMEM for 24 h. The cells were lysed, and reporter activity was measured using the Dual-Luciferase Reporter Assay System (Promega), following manufacturer’s instructions. Firefly luciferase luminescence was normalized to *Renilla* luciferase luminescence.

### Mammalian two-hybrid assays

COS-1 cells were transfected with pGL4.31, PGC1α-LXXLL pFN11A, hPXR-pFN10A, and the *Renilla* luciferase–expressing plasmid phRL-CMV using Lipofectamine 3000. The cells were then treated with vehicle (0.1% or 0.2% DMSO) or drugs in serum-free DMEM for 24 h, and the reporter activity was measured using the Dual-Luciferase Reporter Assay System.

### Statistical analysis

Statistical analyses were conducted using GraphPad Prism (GraphPad Software). The significance of differences was assessed by one-way analysis of variance (ANOVA) followed by Bonferroni’s correction for the comparison of multiple groups data. All experiments were repeated at least twice to confirm reproducibility.

## Data availability

The datasets generated and analyzed in this study are included within the manuscript and supplementary information and can be obtained from the corresponding authors upon reasonable request.

## Supporting information

This article contains supporting information.

## Conflict of interest

The authors declare that they have no conflicts of interest with the contents of this article.
