# Supplementary material for: Helix 12 stabilization contributes to basal transcriptional activity of PXR
Source: J Biol Chem. 2021 Jul 17;297(3):100978. doi: 10.1016/j.jbc.2021.100978 (PMC8390552; doi:10.1016/j.jbc.2021.100978)
Supplement: Supplemental Figures S1–S8 [file mmc1.pdf]

**Supporting information**

# Helix 12 Stabilization Contributes to Basal Transcriptional Activity of PXR

Ryota Shizu[1], Hikaru Nishiguchi[1], Sarii Tashiro[1], Takumi Sato[1], Ayaka Sugawara[1], Yuichiro Kanno[1], Takuomi Hosaka[1], Takamitsu Sasaki[1], Kouichi Yoshinari[1]

[1]Laboratory of Molecular Toxicology, School of Pharmaceutical Sciences, University of Shizuoka, 52-1 Yada, Suruga-ku, Shizuoka 422-8526, Japan

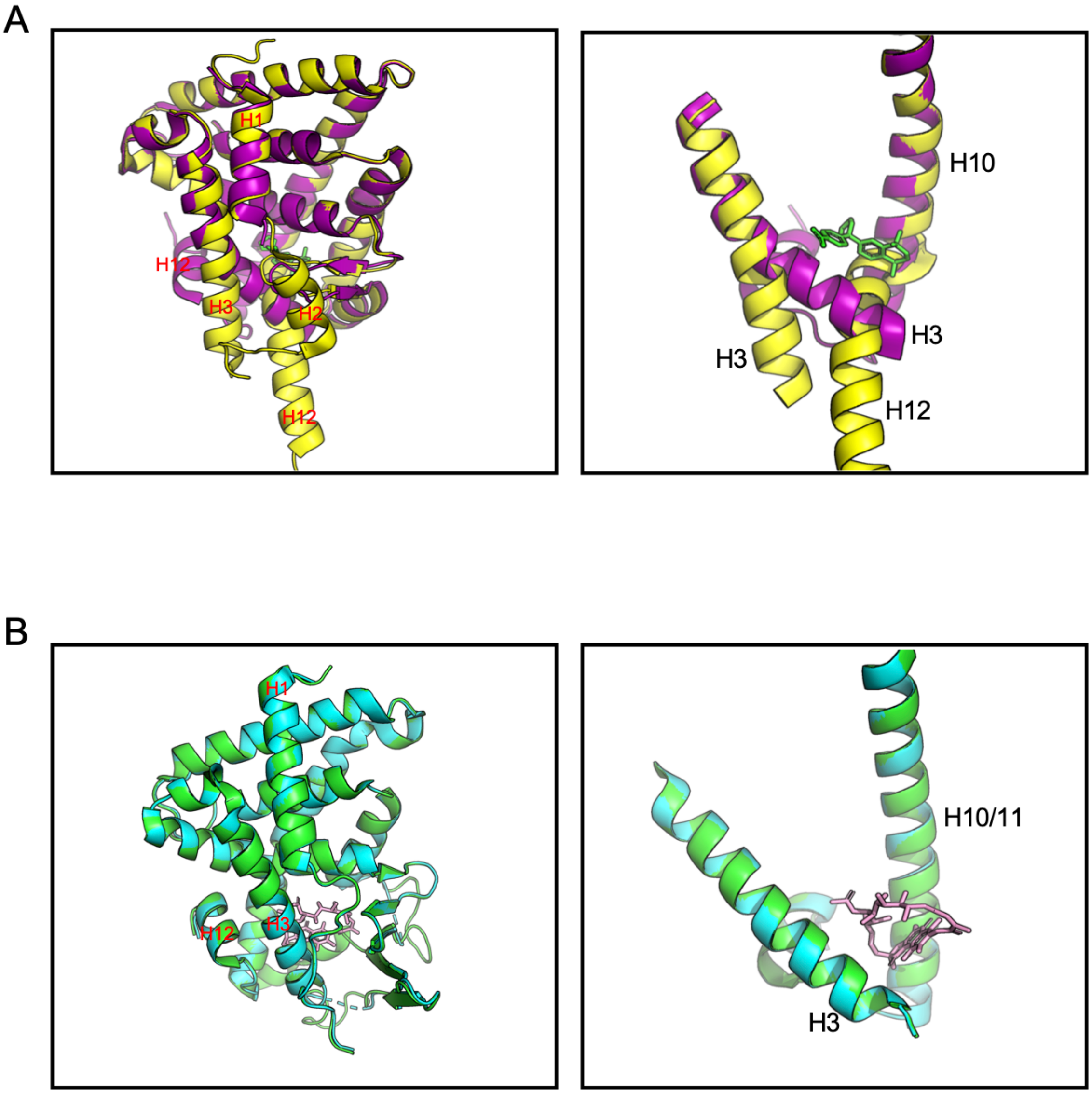


**Fig. S1.** Superimposed crystal structures of (A) RXRα LBD and (B) PXR LBD with or without a ligand shown in Fig. 1 (purple, 1mvc; yellow, 6hn6; cyan, 1skx; green, 1ilg). Left, the entire LBD structures. Right, a close-up view of H3. The N-terminus of H3 in the liganded-RXRα bends to have a close contact with the ligand SR11237.

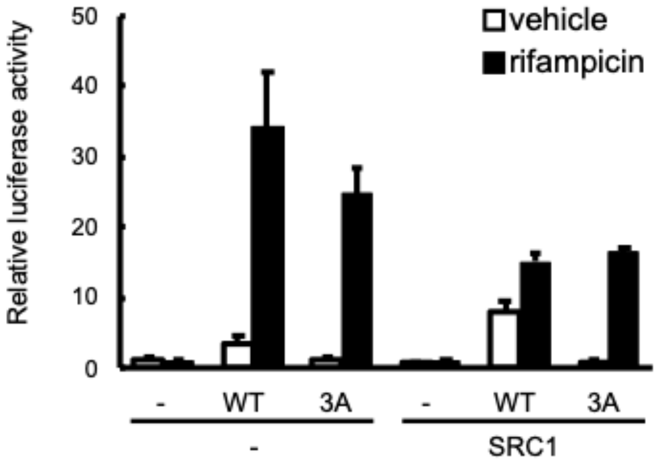


**Fig. S2.** Reporter gene assays were performed in COS-1 cells with the reporter construct containing the promoter for *CYP3A4* (p3A4-pGL3) and an expression plasmid for WT PXR (WT) or PXR-3A (3A) in combination with or without an expression plasmid for SRC1. Cells were treated with rifampicin (10 μM) or vehicle (0.1% DMSO) for 24 h, then reporter activity was determined. Data are shown as the mean of relative activities of four wells in each group to vehicle-treated cells without PXR and SRC1. Error bars represent the standard deviations.

A

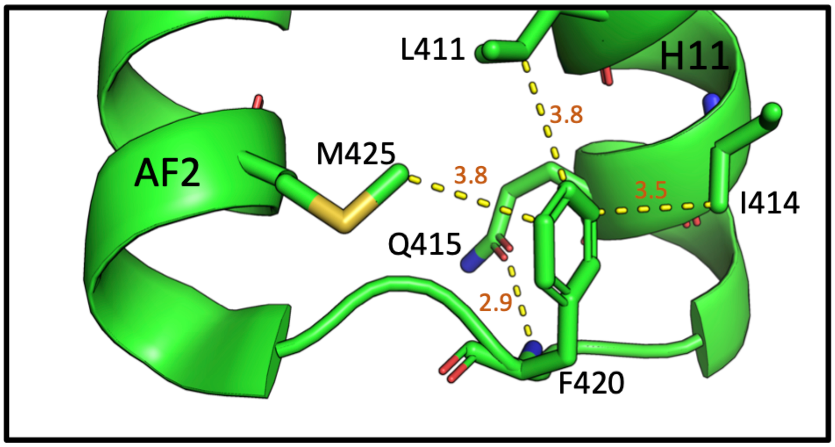


B

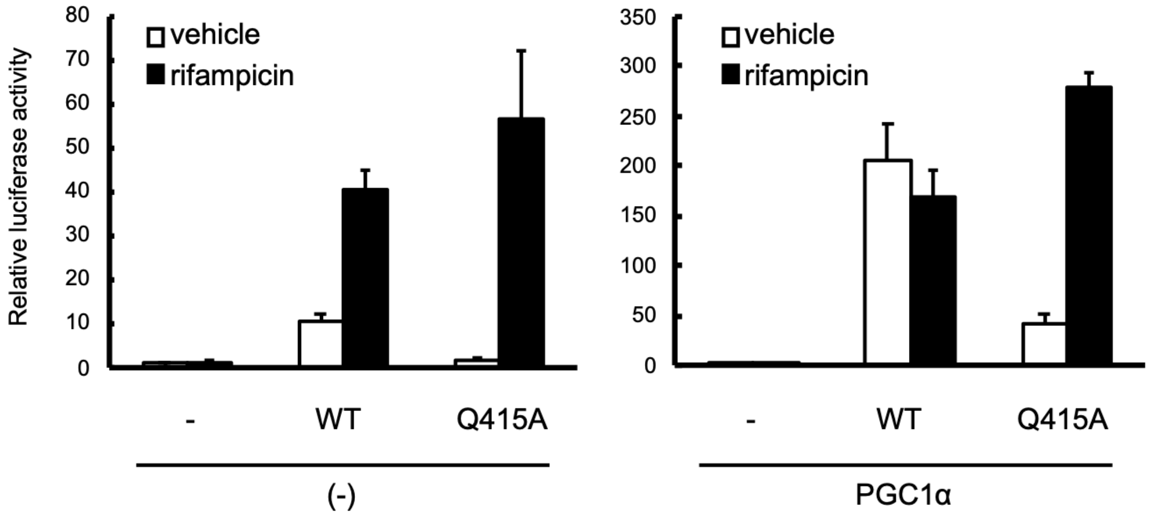


**Fig. S3.** (A) A close-up view of close contact residues in H11–H12 with Phe420 (1skx). (B) Reporter gene assays were performed in COS-1 cells with the reporter construct containing the promoter for *CYP3A4* (p3A4-pGL3) and expression plasmids for either WT PXR (WT) or PXR-Q415A (Q415A). Cells were treated with rifampicin (10 μM) or vehicle (0.1% DMSO) for 24 h, and then reporter activity was determined. Data are shown as the mean of relative activities of four wells in each group to vehicle-treated cells without PXR. Error bars represent the standard deviations.

A

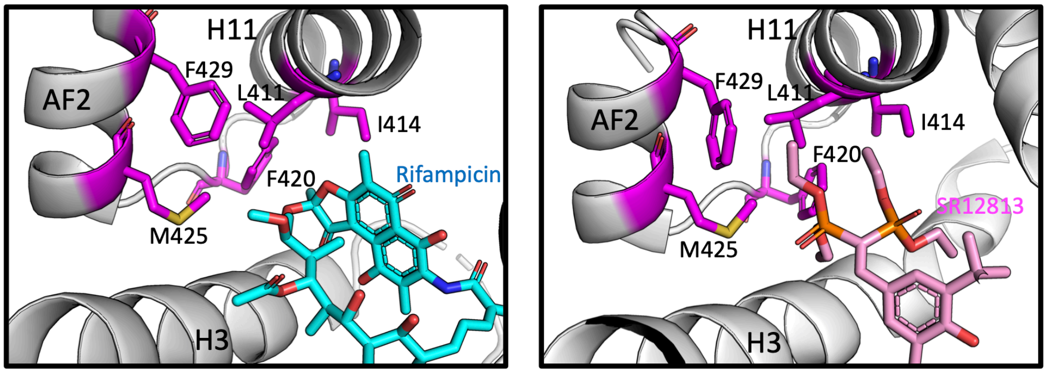


B

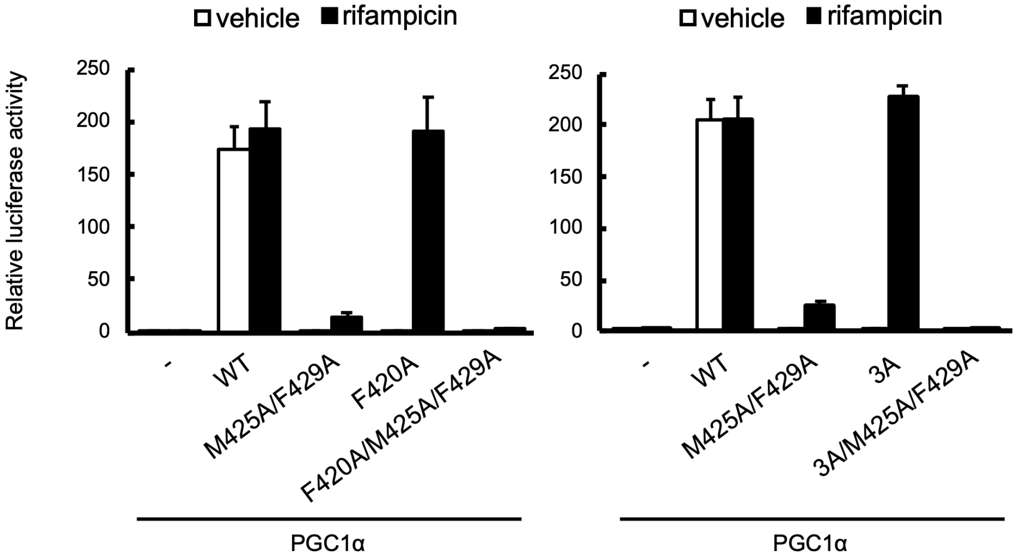


**Fig. S4.** (A) Close-up views of the contact of residues in PXR H11–H12 with ligands (rifampicin; 1skx; SR12813, 1nrl). (B) Reporter gene assays were performed in COS-1 cells with the reporter construct containing the promoter for *CYP3A4* (p3A4-pGL3) and an expression plasmid for WT PXR (WT), PXR-M425A/F429A (M425A/F429A), PXR-F420A (F420A), PXR-F420A/M425A/F429A (F420A/M425A/F429A), PXR-3A (3A), or PXR-3A/M425A/F429A (3A/M425A/F429A) in combination with PGC1α. Cells were treated with rifampicin (10 μM) or vehicle (0.1% DMSO) for 24 h, and then reporter activity was determined. Data are shown as the mean of relative activities of four wells in each group to vehicle-treated cells without PXR and PGC1α. Error bars represent the standard deviations.

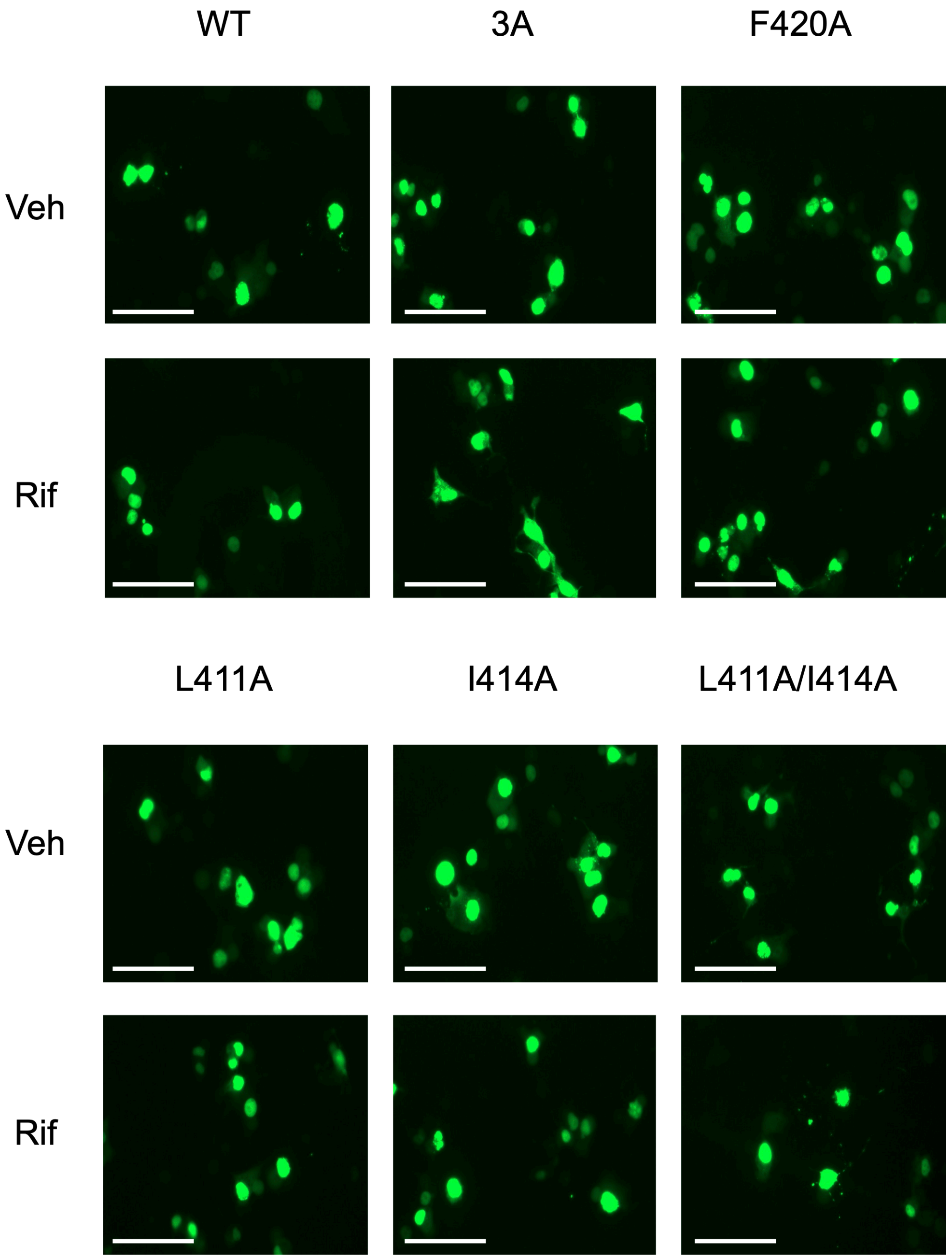


**Fig. S5.** COS-1 cells were transfected with expression plasmids for GFP-tagged WT PXR (WT), PXR-3A (3A), PXR-F420A (F420A), PXR-L411A (L411A), PXR-I414A (I414A), or PXR-L411A/I414A (L411A/I414A). Cells were treated with rifampicin (10 μM) or vehicle (0.1% DMSO) for 24 h, and then cellular distribution was assessed by fluorescence microscopy. The scale bar indicates 100 μm.

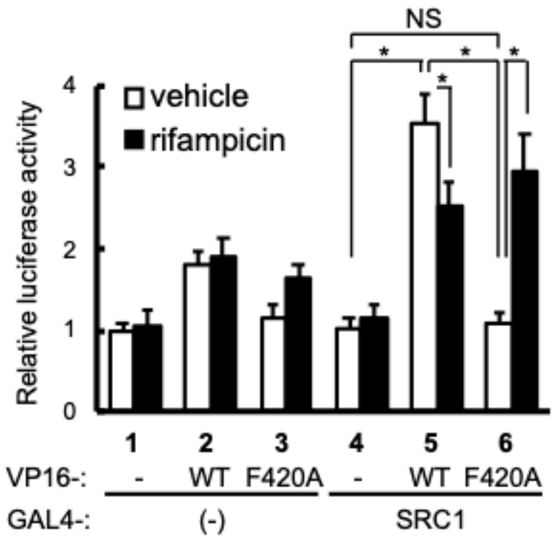


**Fig. S6.** Mammalian two-hybrid assays were performed in COS-1 cells with pGL4.31, pFN11A expressing GAL4 or GAL4 fused with SRC1, and pFN10A expressing VP16 or VP16 fused with WT PXR (WT) or PXR-F420A (F420A). Cells were treated with vehicle (0.1% DMSO) or rifampicin (10 μM) for 24 h, then the reporter activity was determined. Data are shown as the mean of relative activities of four wells to vehicle-treated cells without PXR and PGC1α. Error bars represent the standard deviations. Statistical analyses were performed for the indicated combinations with Bonferroni's correction (*$p < 0.05$; NS, not significant).

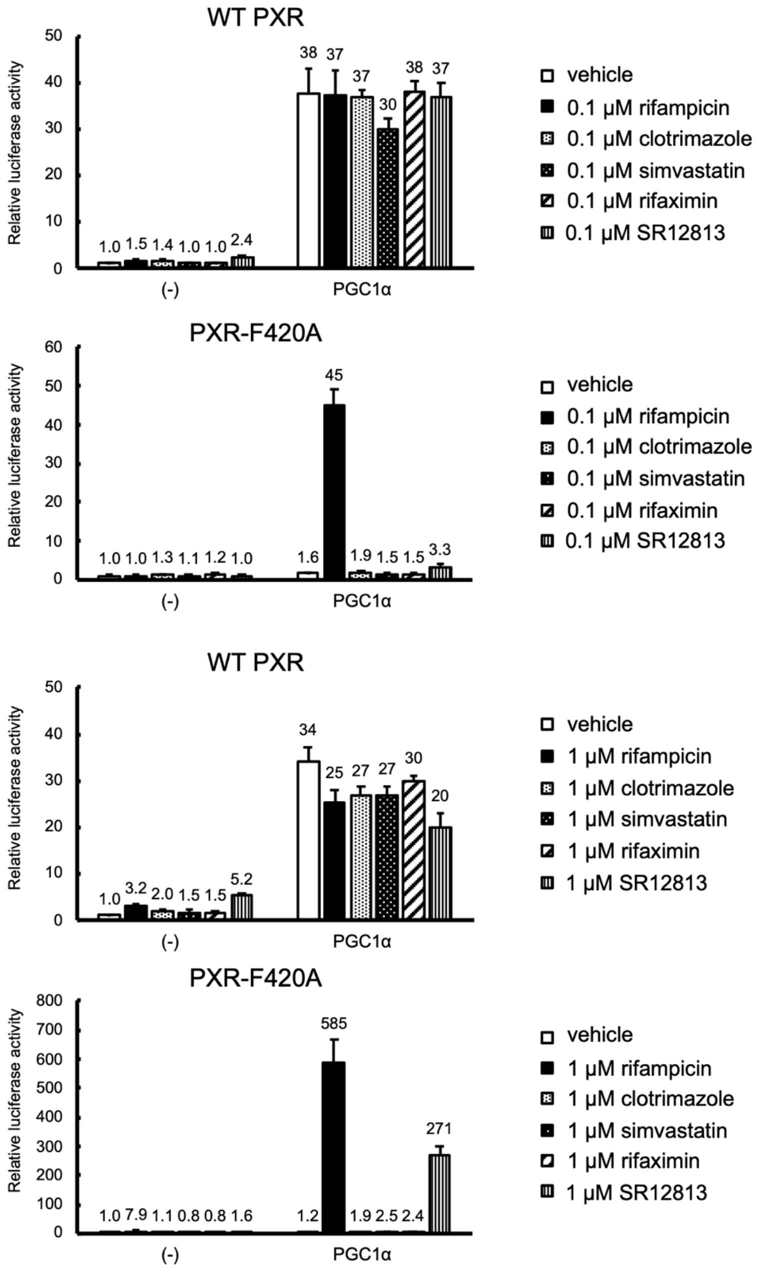


**Fig. S7.** Reporter gene assays were performed with the ligands at 0.1 and 1 μM as in Fig. 4. Data are shown as the mean of relative reporter activities of four wells in each group to that of vehicle-treated cells without PGC1α. Error bars represent the standard deviations. The numbers above the column indicate the relative reporter activity to that of vehicle-treated cells without PGC1α expression.

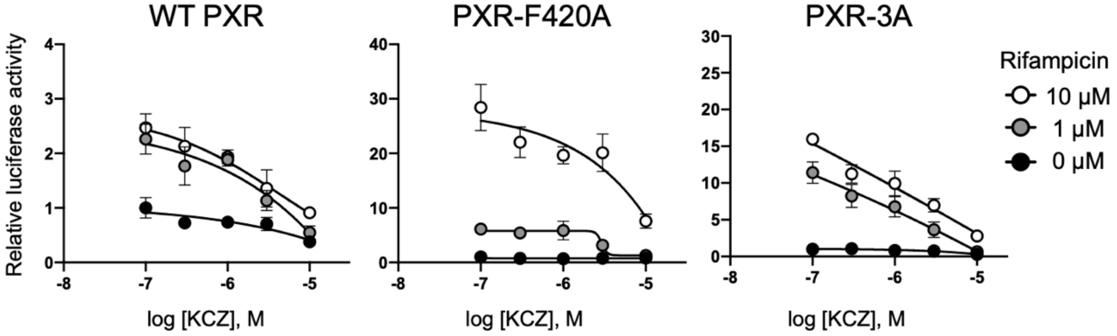


**Fig. S8.** Reporter gene assays were performed in COS-1 cells with the reporter construct containing the promoter for *CYP3A4* (p3A4-pGL3) and the expression plasmid for WT PXR, PXR-F420A, or PXR-3A in combination with the PGC1α expression plasmid. Cells were treated with rifampicin and/or ketoconazole (KCZ) at the indicated concentrations for 24 h, and then the reporter activity was determined. Data are shown as the relative reporter activity to vehicle-treated cells and each point represents the mean of relative activities of four wells ± the standard deviations.
